# Cognitive- and memory-enhancing effects of Augmentin in Alzheimer’s rats through regulation of gene expression and neuronal cell apoptosis

**DOI:** 10.3389/fphar.2023.1154607

**Published:** 2023-03-09

**Authors:** Mahmoud Kandeel, Mohamed A. Morsy, Hany M. Abd El-Lateef, Mohamed Marzok, Hossam S. El-Beltagi, Khalid M. Al Khodair, Ibrahim Albokhadaim, Katharigatta N. Venugopala

**Affiliations:** ^1^ Department of Biomedical Sciences, College of Veterinary Medicine, King Faisal University, Al-Ahsa, Saudi Arabia; ^2^ Department of Pharmacology, Faculty of Veterinary Medicine, Kafrelsheikh University, Kafrelsheikh, Egypt; ^3^ Department of Pharmaceutical Sciences, College of Clinical Pharmacy, King Faisal University, Al-Ahsa, Saudi Arabia; ^4^ Department of Pharmacology, Faculty of Medicine, Minia University, El-Minia, Egypt; ^5^ Department of Chemistry, College of Science, King Faisal University, Al-Ahsa, Saudi Arabia; ^6^ Department of Chemistry, Faculty of Science, Sohag University, Sohag, Egypt; ^7^ Department of Clinical Sciences, College of Veterinary Medicine, King Faisal University, Al-Ahsa, Saudi Arabia; ^8^ Department of Surgery, Faculty of Veterinary Medicine, Kafrelsheikh University, Kafrelsheikh, Egypt; ^9^ Agricultural Biotechnology Department, College of Agriculture and Food Sciences, King Faisal University, Al-Ahsa, Saudi Arabia; ^10^ Biochemistry Department, Faculty of Agriculture, Cairo University, Giza, Egypt; ^11^ Department of Anatomy, College of Veterinary Medicine, King Faisal University, Al-Ahsa, Saudi Arabia; ^12^ Department of Biotechnology and Food Science, Faculty of Applied Sciences, Durban University of Technology, Durban, South Africa

**Keywords:** Alzheimer’s disease, memantine, augmentin, expression, apoptosis

## Abstract

**Introduction:** Alzheimer’s disease (AD) is the most common type of dementia among older persons. This study looked at how Augmentin affected behavior, gene expression, and apoptosis in rats in which AD had been induced by scopolamine.

**Methods:** The rats were divided into five groups: control, sham, memantine, Augmentin, and pre-Augmentin (the last group received Augmentin before scopolamine administration and was treated with memantine). A Morris water maze was utilized to measure spatial memory in the animals, and real-time quantitative reverse transcription PCR (qRT-PCR) and flow cytometry were employed to analyze gene expression and neuronal cell apoptosis, respectively.

**Results:** Memantine and Augmentin increased spatial memory in healthy rats. The use of scopolamine impaired spatial memory. Both Augmentin and memantine improved spatial memory in AD rats, particularly in the group that received memantine; however, the outcomes were more substantial when Augmentin was administered before scopolamine was given to induce AD. Furthermore, the expression of presenilin-2 (PSEN2) and inositol-trisphosphate 3-kinase B (ITPKB) increased, whereas the expression of DEAD-box helicase 5 (DDX5) fell in the AD-treated groups; however, the results were more substantial after combination therapy. According to flow cytometry studies, Augmentin pre-treatment reduced apoptosis in AD rats.

**Discussion:** The results showed that administering Augmentin to AD rats before memantine improved their spatial memory, reduced neuronal cell death, upregulated protective genes, and suppressed genes involved in AD pathogenesis.

## 1 Introduction

Neurological disorders are major hazards for humans. A report from the World Health Organization indicated that neurological disorders affect about a billion human beings worldwide and that one of these, Alzheimer’s disease (AD), affects 24 million people (WHO, 2007). There are approximately 6.8 million annual deaths attributable to neurological illnesses (WHO, 2007). AD is a type of brain disease that typically affects older adults. Clinically, it is a neurodegenerative condition that progresses and is marked by a deterioration in behavior and cognitive function (Kumar et al., 2018). In some parts of the brain, the production and necrosis of brain cells, as well as the formation of spherical protein complexes known as aging plaques and neurofibrillary tangles in the neuron’s core (Zvěřová, 2019). Genetic factors play important roles in the pathogenesis of AD. For instance, various types of triggering receptors expressed on myeloid cell 2 (TREM2) genes are considered predisposing factors for AD (Singh et al., 2019a).

Aducanumab is the only drug that has been licensed for use in the treatment of AD (Padda and Parmar, 2022). The drug is a human antibody utilized as an immunotherapy treatment with the purpose of reducing amyloid plaques, which are brain lesions associated with AD. Brain-derived neurotrophic factors (BDNFs) and transcription factors are valid targets in the fight against AD (Rai et al., 2021; Gao et al., 2022). A correlation has been established between low levels of BDNF and the buildup of amyloid-β (Aβ), neuroinflammation and neuronal cell death. The protective effect of some medicinal plants and their alkaloids against AD is now undergoing investigation (Singh et al., 2019b; Singh et al., 2021).

Studies have shown that the expression of some genes, such as presenilin-2 (PSEN2), DEAD-box helicase 5 (DDX5), and inositol-trisphosphate 3-kinase B (ITPKB), is dysregulated in patients with AD (Kar et al., 2011; Stygelbout et al., 2014; Lanoiselee et al., 2017). PSEN2 was shown to be significantly downregulated in the auditory cortex of AD patients, and PSEN2 expression changes could be a marker of AD (Delabio et al., 2014). A single mutation in the PSEN2 gene has been associated with the early onset of AD (Giau et al., 2018). ITPKB mRNA has been found to be significantly increased in AD (Stygelbout et al., 2014). In cell cultures, the overproduction of Aβ peptides and an increase in cell deaths were both linked to ITPKB overexpression (Stygelbout et al., 2014). DDX5 deficiency has been connected to the pathogenic characteristics of AD in terms of interference with a range of biological processes, including protein splicing, cell proliferation, transcription, RNA unwinding, and disturbances in energy metabolism (Li et al., 2021).

AD patients receive either disease-modifying or symptomatic medications. All medications licensed for AD treatment alter neurotransmitters such as glutamate and acetylcholine. These medications postpone the onset of the disease and slow its progression (Yiannopoulou and Papageorgiou, 2013). Memantine is one of a class of drugs known as N-methyl-D-aspartate receptor (NMDA) antagonists, which are thought to function by blocking NMDA receptors on ion channels (Olivares et al., 2012). In a variety of animal models, memantine has been proven to protect against neurodegenerative and vascular processes. Memantine, a partial NMDA receptor antagonist, works to balance the glutamatergic system and relieve cognitive and memory difficulties caused by excess glutamate. Memantine’s low affinity and fast off-rate kinetics at the NMDA receptor-channel level preserve the receptor’s physiological function, which adds to the drug’s high tolerance and low adverse event profile (Olivares et al., 2012).

Augmentin is the trade name for amoxicillin/clavulanic acid, which contains a *ß*-lactam antibiotic and *ß*-lactamase inhibitor (Gillies et al., 2015). Augmentin has widely been used to treat bacterial diseases (Lopes-de-Campos et al., 2019). Ampicillin has been investigated as a neuroprotective agent, and it protected neurons from the damaging effects of ischemic brain damage in a dose-dependent manner (Lee et al., 2016). Inducing the glutamate transporter 1 (GLT-1) protein and decreasing the activity of matrix metalloproteinase (MMP) in the mouse hippocampus are the two mechanisms through which ampicillin provides protection against ischemia‒reperfusion brain injury (Lee et al., 2016). It has been demonstrated that the psychoactive substance clavulanic acid can change how the central nervous system functions. Importantly, clavulanic acid has been shown to enhance motor function in animal models of neurotoxic intoxication, suggesting that it may be a neuroprotective agent (Huh et al., 2010). Additionally, it was demonstrated that clavulanic acid shields nerve cells against degeneration (Kost et al., 2012). Dopaminergic cell survival is improved by clavulanic acid’s anti-apoptotic properties. The loss of mitochondrial membrane potential and the generation of reactive oxygen species (ROS) generated by neurotoxins are prevented by clavulanic acid (Kost et al., 2012).

Apoptosis contributes to the significant neuronal loss that occurs in AD patients. There is a decline in attention and other cognitive capacities as a result of the destruction or death of hippocampal cells (Obulesu and Lakshmi, 2014). Apoptosis contributes to the pathogenesis of AD, while abnormal apoptotic pathways can be regarded as a major cause of the observed pathological features of AD. The pathological hallmarks of AD, which are Aβ plaques, mitochondrial dysfunction, generation of ROS, and oxidative damage, all contribute to an aberrant apoptotic cascade (Sharma et al., 2021). Several pathways, such as phosphatidylinositol-3-kinase–protein kinase B (PI3K/PKB), mitogen-activated protein kinases (MAPKs), and the mammalian target of rapamycin (mTOR) signaling, are modulated during apoptosis in AD (Sharma et al., 2021).

The application of scopolamine, a tropane alkaloid having antagonistic actions on muscarinic receptors, has been shown to cause alphameric-like conditions in rats by interrupting the cholinergic neurotransmitter system and causing memory impairment. It has also been shown that hippocampus cells undergo apoptosis after scopolamine administration (Lochner and Thompson, 2016). For this reason, rats have been given scopolamine and utilized as animal models to investigate AD and anti-dementia medications (Chen et al., 2019). This study investigated the potential additive interactions of Augmentin on memantine-induced relief of scopolamine-induced memory impairment in rats. The expected outcome of this work was to establish new drug combinations with high efficiency in handling AD. We aimed to investigate the role of Augmentin on AD’s pathogenesis in combination with a routine treatment to determine the possible prophylactic and therapeutic effects of Augmentin in improving memory and cognitive function in the AD rat model.

## 2 Materials and methods

### 2.1 Rat model of AD and drug administration

The rationale for this study was to investigate the neuroprotective effects and mechanisms of the actions of Augmentin in AD by using a rat AD model. For this purpose, five groups of rats were allocated to provide experimental controls and AD rats with or without treatment with Augmentin and/or memantine.

In this study, 50 adult male rats weighing approximately 200 g were used. They were given water and food and preserved in optimum conditions. The animals were handled and treated according to the animal handling guidelines. The protocol was approved by the ethics committee, approval number KFU-REC-2023-JAN-ETHICS475. The rats were divided into five groups. The rats in the control group were left untreated, while the rats in the sham group received 0.1 mL of standard saline intraperitoneally (IP). The third to fifth groups received scopolamine (2 mg/kg) injected IP to induce AD. The third group received memantine (10 mg/kg) IP. The fourth group of rats was injected with 10 mg/kg of Augmentin. The fifth group of rats was injected with 10 mg/kg of Augmentin IP before scopolamine administration, and was labelled the pre-Augmentin group.

### 2.2 Behavioral research

The Morris water maze is a standard device used to investigate learning and memory in rat experiments (D’Hooge and De Deyn, 2001). The experimental setup and procedures were as previously described (D’Hooge and De Deyn, 2001; Nunez, 2008; Callaway et al., 2012). After treatment, the rats were placed in the Morris water maze for 1 min daily for 3 days. Rats that could find the Q2 area were allowed to stay 5 s longer. If a rat did not find the platform during this time, it was manually guided to it to stay on it and remember its location. Each rat was trained only once a day. On the third day, rats were randomly placed in different areas of the Morris water maze, and the platform’s position (in area Q2) was excluded. The percentage of time rats spent in Q2 was noted.

### 2.3 Separation of the hippocampus from rat brain

After the rat was sacrificed under standard procedures, the brain was taken from the skull and washed in ice-cold Milli Q water that had undergone DEPC treatment (Chen et al., 2013). The right and left hemispheres of the removed brain were separated by cutting it in half on a metal plate. The ventral half of the brain was lifted so that the midbrain’s hippocampus could be accessed to collect it. Then, two Dumont No. 5 forceps were used to separate the hippocampus from the cortex. The sample was immediately placed in liquid nitrogen and kept at minus 80°C.

### 2.4 Real-time PCR

The rats’ brains were taken out when the behavioral tests were finished and processed by means of RT-PCR as previously described with slight modifications (Chen et al., 2013; Zhu et al., 2014). The total RNA was extracted utilizing TIANGEN BIOTECH (Beijing, China) following the manufacturer’s instructions. The reverse transcription of the RNAs into cDNAs was then completed using a cDNA Synthesis kit from Invitrogen (Waltham, Massachusetts, United States). Takara Bio’'s SYBR Premix Ex Taq in QuantStudio 5 Real-Time PCR (Thermo Fisher Scientific, Waltham, Massachusetts, United States) instrument was used to conduct the real-time PCR reaction. The internal control was the amount of beta-actin mRNA. Primer sequences are shown in Table 1.

**TABLE 1:** Primer pairs and their nucleotide sequences.

ame	Sequences	
PSEN2	Forward	5′CTG​GTG​TTC​ATC​AAG​TAC​CTG​CC3′
	Reverse	5′TTC​TCT​CCT​GGG​CAG​TTT​CCA​C3′
DDX5	Forward	5′AGA​GGT​CAC​AAC​TGT​CCA​AAA​CC3′
	Reverse	5′CCA​ATC​CAC​TGA​GAG​CAA​CTG​G3′
ITPKB	Forward	5′ACG​CTA​CAA​CCA​GAT​GGA​CGA​C3′
	Reverse	5′ATG​TCC​TTC​CGC​AAG​CTA​GGC​T3′
Beta actin	Forward	5′CAT​TGC​TGA​CAG​GAT​GCA​GAA​GG3′
	Reverse	5′TGC​TGG​AAG​GTG​GAC​AGT​GAG​G3′

### 2.5 Annexin-PI assay and flow cytometry

The percentage of hippocampal cell apoptosis was determined at this stage using eBioscience Annexin V Apoptosis Detection Kits (Thermo Fisher Scientific, Waltham, Massachusetts, United States) as previously described (Zhao et al., 2013; Fang et al., 2017). In brief, cells were resuspended in Annexin-V binding buffer, and 100 L of cell suspensions and 5 L of each FITC Annexin V and PI staining solution were added and left at room temperature for 20 min. The apoptosis rate was measured after adding Annexin-V binding buffer using a BD FACSCantoTM Flow Cytometry System; it was analyzed with Flowjo software.

### 2.6 Statistical analysis

A one-way analysis of variance (ANOVA) was used to compare the results, and a *post hoc* test was used to determine significant differences between the groups. All tests were considered significant if the *p*-value was less than .05.

## 3 Results

### 3.1 The effect of memantine and Augmentin on the spatial memory of healthy rats

**FIGURE 1:**
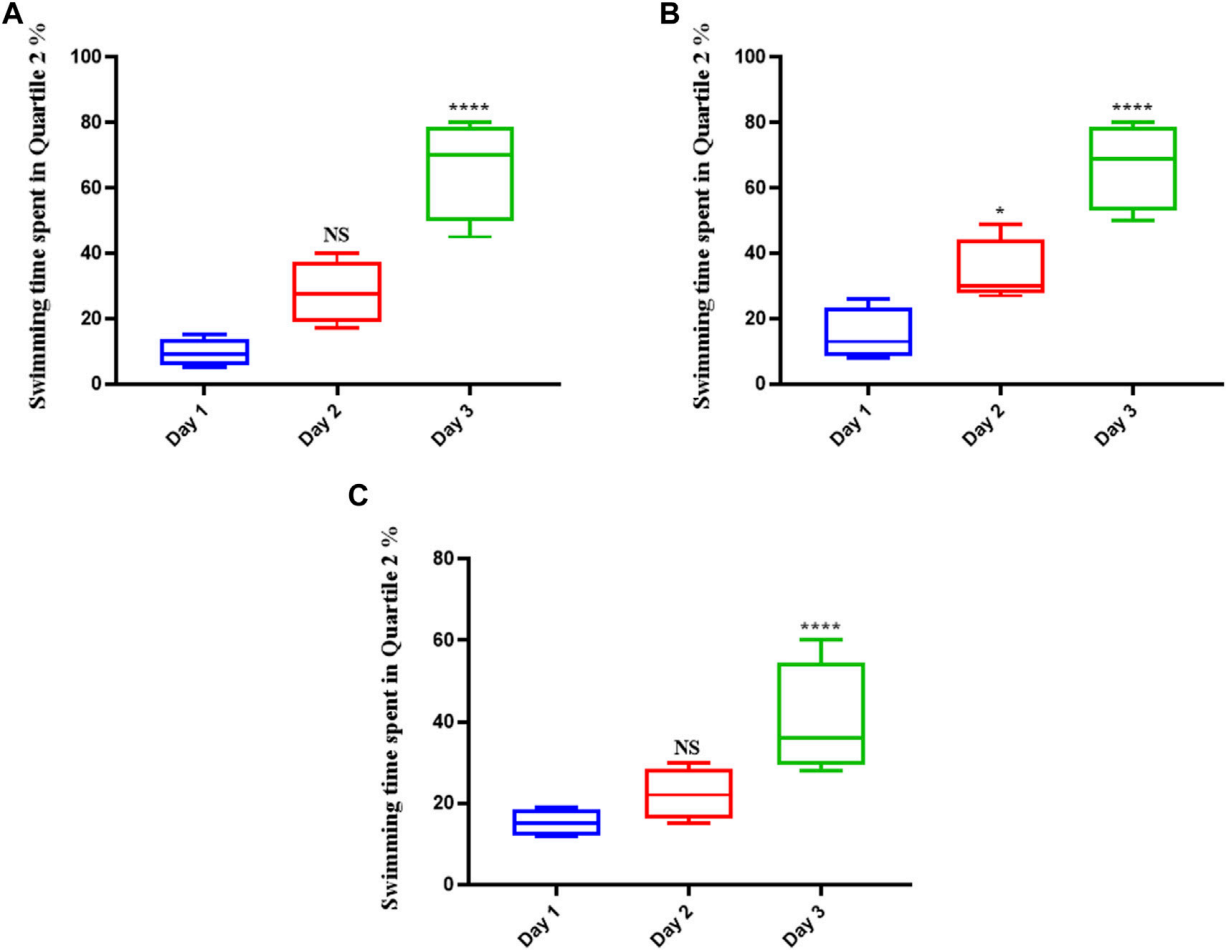
The effect of Memantine and Augmentin on healthy rats’ spatial memory. The results from the control group **(A)** as well as the Memantine **(B)**- and Augmentin **(C)**-receiving groups revealed that the rats swam more in the platform position in the subsequent days of the experiment by determining the spatial position of the platform in the maze. (NS, non-significant; *p < 0.05, and ****p < 0.0001).

The rats in the control group (which were given normal saline) swam more in the platform position as the trial progressed, suggesting that they had learned to navigate the maze and had memorized the platform’s precise location (Figure 1A). They remained in that position until the experiment was over if they could see the platform. Memantine recipients had the same outcomes as the control group over time (see Figure 1B). Similarly, the rats treated with Augmentin had increased swimming times as the experiment progressed (see Figure 1C). This result indicated that the learning circuits and spatial memory of the control group rats as well as the rats treated with memantine and Augmentin were in fine working order.

### 3.2 The effect of scopolamine on spatial memory in rats

**FIGURE 2:**
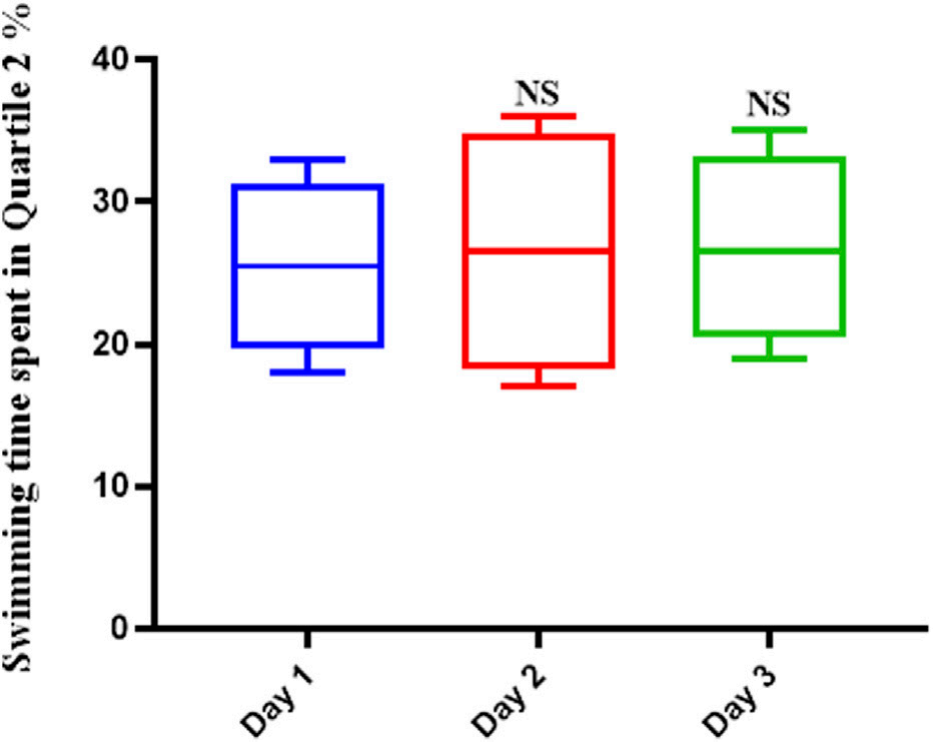
Scopolamine impairs spatial memory in rats. The average swimming duration of Scopolamine-treated rats did not change throughout the experiment, indicating that Scopolamine inhibited these animals’ learning mechanisms (NS: Not significant).

Scopolamine hindered the learning mechanisms of the rats tested. This was shown by the fact that the average swimming time did not change over the course of the 3-day trial or on the final test day (Figure 2). The average swimming time did not show significant differences over the duration of the trial.

### 3.3 The effect of memantine treatment on spatial memory in rats

**FIGURE 3:**
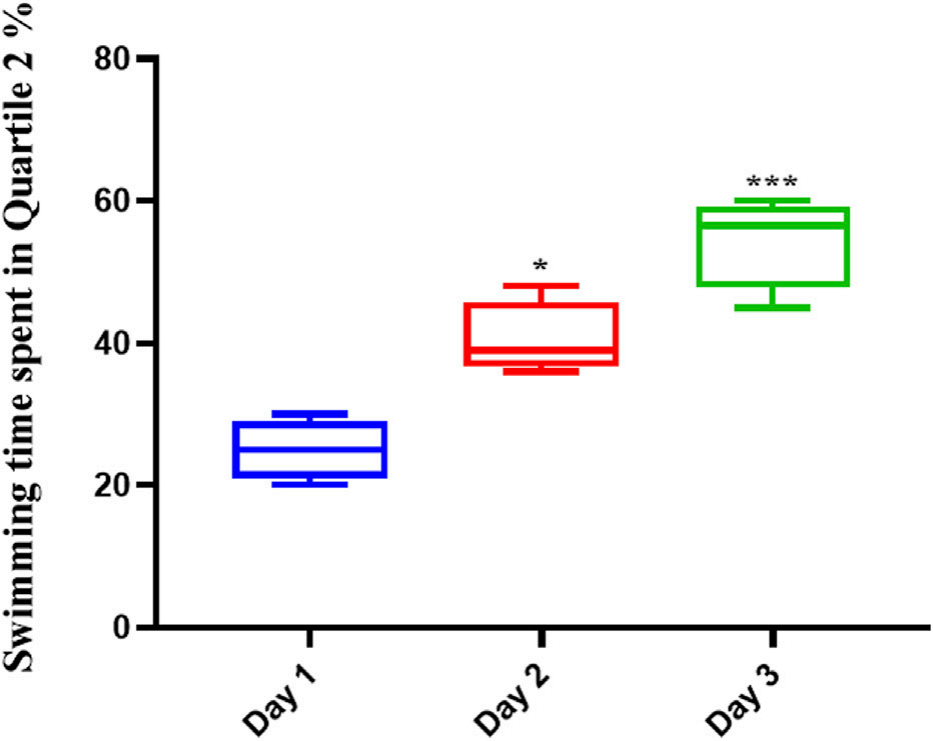
Memantine improved spatial memory in Scopolamine-induced Alzheimer’s rats. The results showed that Memantine improved Scopolamine-injected AD rats’ spatial memory, as evidenced by increased swimming time in the Q2 position during the experiment (*p < 0.05, ***p < 0.001).

Memantine improved the spatial memory in scopolamine-injected AD rats (Figure 3), allowing them to recall the spatial position of the platform; this was evidenced by their increased swimming time in the Q2 position during the second (*p* < .01) and third (*p* < .0001) days of the experiment.

### 3.4 The effect of pre-treatment with Augmentin before scopolamine or memantine in AD rats

**FIGURE 4:**
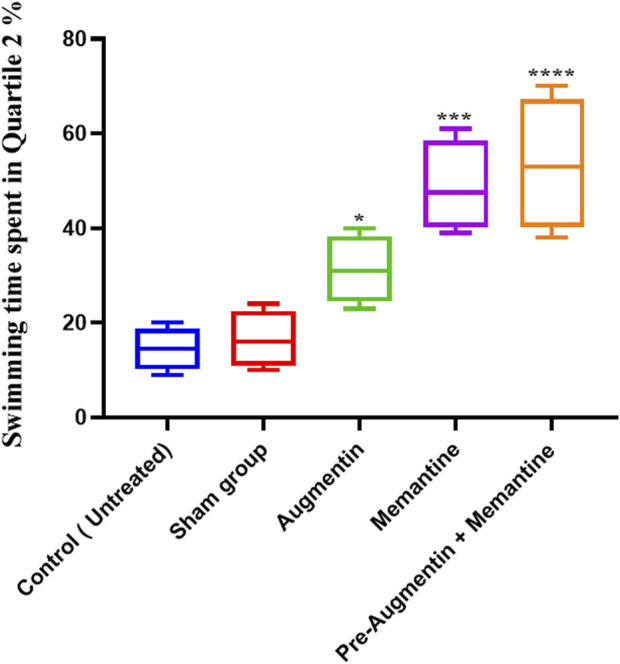
The effect of Memantine, Augmentin, and Pre-Augmentin therapy before Memantine administration on spatial memory in Scopolamine-induced Alzheimer’s rats on the day of the experiment. In the experiment, increased swimming time in the Q2 position was observed in all three groups; however, it was more significant when Augmentin was administered before Memantine (*p < 0.05, ***p < 0.001).

The results showed that administration of Augmentin before scopolamine injection (AD induction) followed by administration of memantine had a more significant effect than the administration of memantine alone. On the day of the experiment (Figure 4), compared with the non-treated control group, the AD rat groups receiving Augmentin (*p* < .01), memantine (*p* < .0001), or pre-treatment with Augmentin (*p* < .0001) had increased swimming times.

**FIGURE 5:**
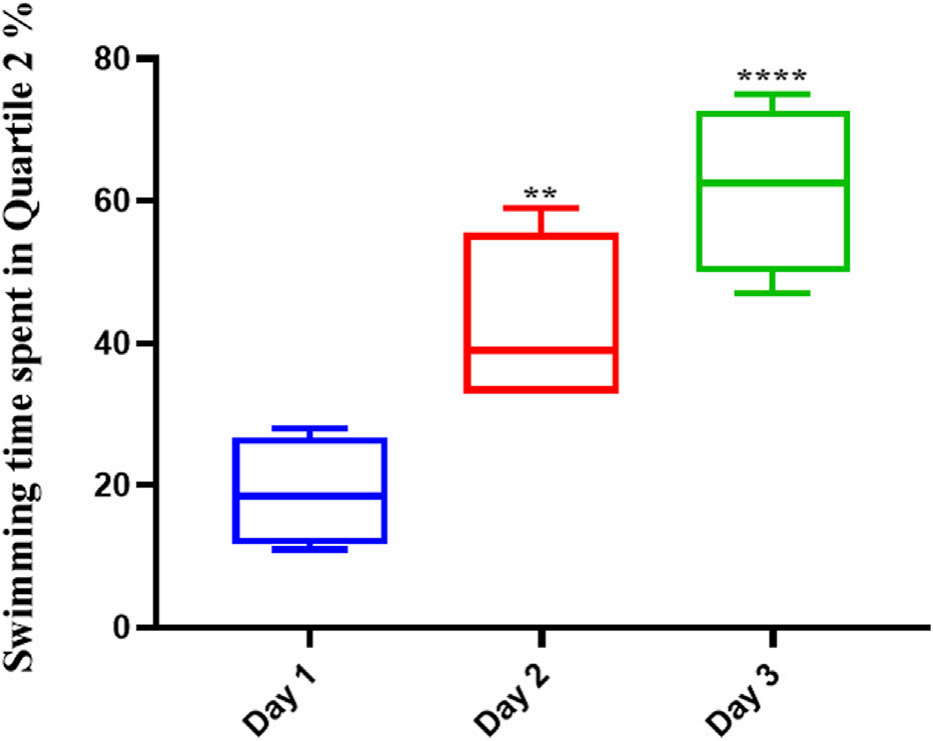
Pre-treatment with Augmentin improved the effect of Memantine in Alzheimer’s rats. This graph shows that administering Augmentin before AD induction and then Memantine increases the average swimming time during the experiment (**p < 0.01 and ****p < 0.0001).

The average swimming time of the animals during the experiment for the pre-Augmentin group showed a significant increase on the second (*p* < .01) and third (*p* < .0001) days of the experiment (Figure 5). The results for rats given pre-treatment Augmentin were insignificant during the investigation (i.e., data are not shown).

### 3.5 Gene expression

**FIGURE 6:**
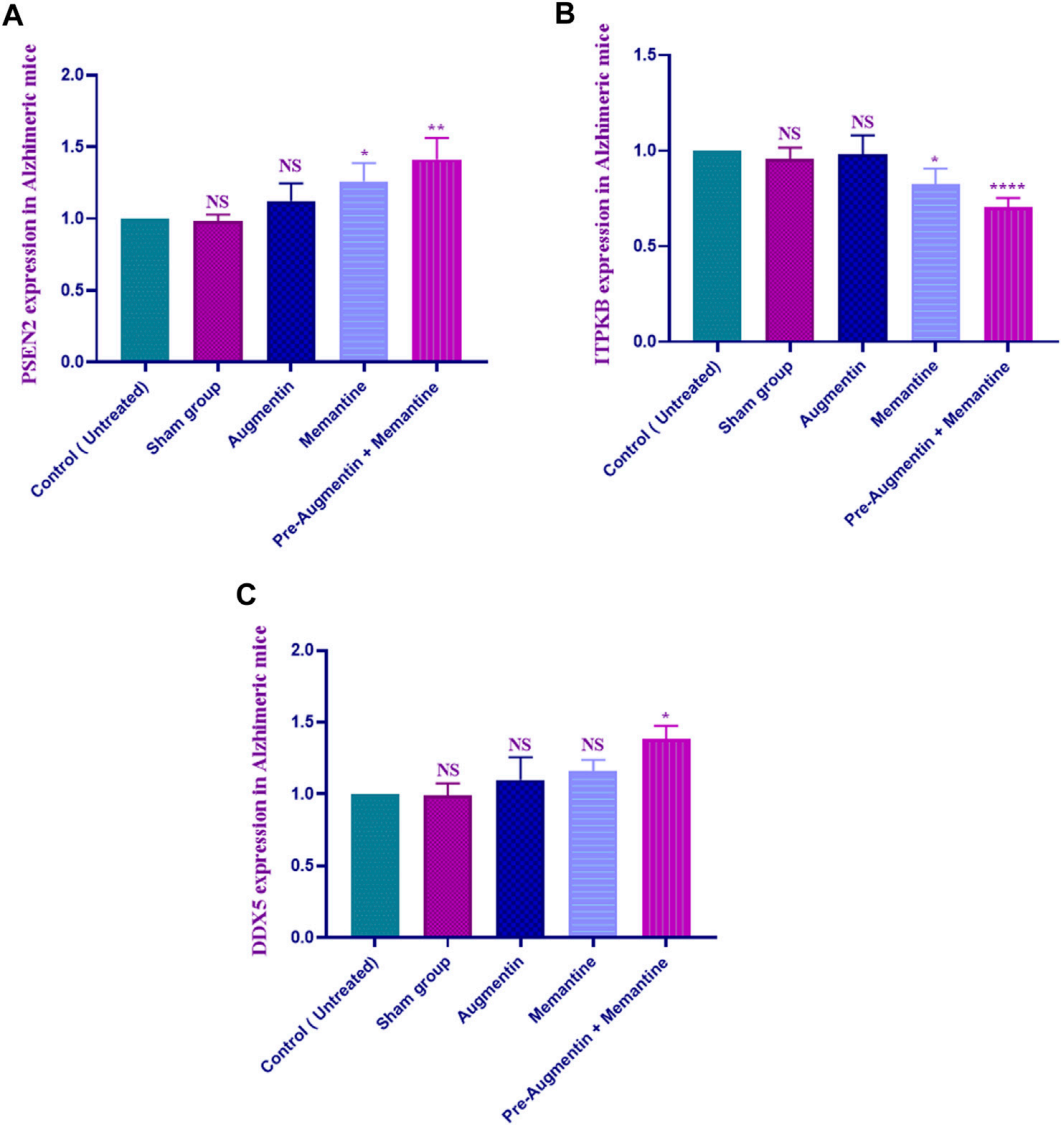
Augmentin pre-treatment enhanced Memantine’s effect on AD-related genes. These graphs show that Augmentin pre-treatment increased PSEN2 **(A)** in Scopolamine-induced Alzheimer’s rats. Augmentin pre-treatment decreased ITPKB **(B)** in Scopolamine-induced Alzheimer’s rats. The expression level of ITPKB **(C)** remained unchanged. Augmentin pre-treatment, on the other hand, produces more significant results in Alzheimer’s rats receiving Memantine (NS, Not significant; *p < 0.05, **p < 0.01, and ****p < 0.0001).

The qRT-PCR results showed that memantine (*p* < .05) and pre-treatment with Augmentin (*p* < .01) significantly increased PSEN2 expression in scopolamine-induced AD rats (Figure 6A), while ITPKB expression showed a decreasing trend in the AD rats receiving memantine (*p* < .05) and pre-treatment with Augmentin (*p* < .0001) (see Figure 6B). However, the expression level of DDX5 remained insignificantly changed. Memantine alone caused a non-significant increase in DDX5 expression. Interestingly, Augmentin pre-treatment in AD rats receiving memantine led to a significant rise in DDX5 expression (*p* < .05) (Figure 6C).

### 3.6 Flow cytometry

**FIGURE 7:**
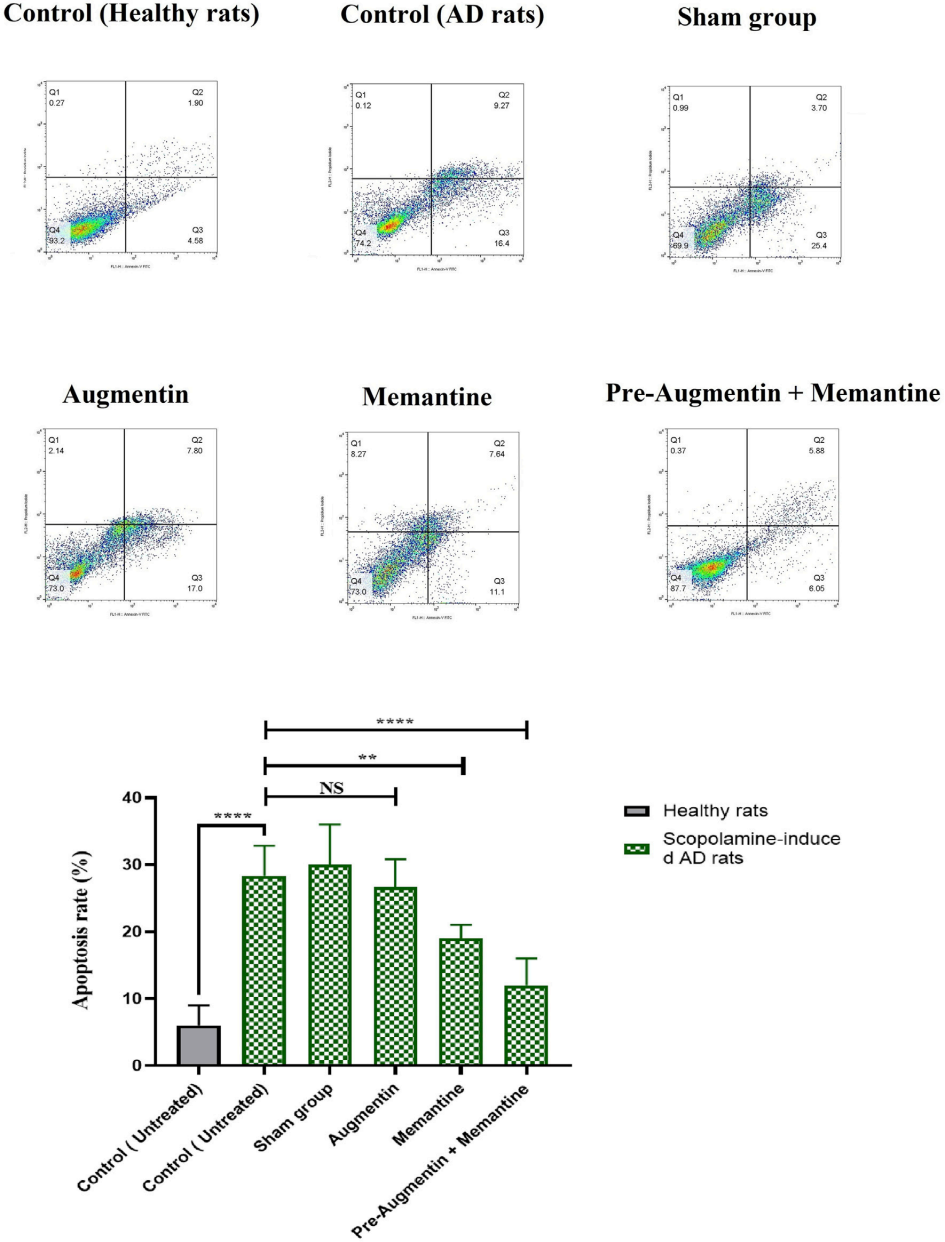
Pre-treatment with Augmentin reduced Scopolamine-induced hippocampal cell apoptosis. Flow cytometry results showed that Augmentin pre-treatment reduced Scopolamine-induced cell death in Alzheimer’s rats given Memantine; however, the results for the group that only received Augmentin were insignificant (NS, Not significant; **p < 0.01, and ****p < 0.0001).

The results obtained from flow cytometry showed that Augmentin pre-treatment alleviated scopolamine-induced cell death in AD rats that received memantine; however, the results for the group that received Augmentin alone were insignificant (Figure 7). Therefore, a significant decrease in the rate of apoptosis of neuronal cells was found in the rat groups that received memantine (*p* < .01) or pre-treatment with Augmentin (*p* < .0001).

## 4 Discussion

The effects of Augmentin on gene expression, apoptosis, and behavior in scopolamine-induced AD rats were investigated in this study. Spatial memory was improved in normal rats by injections of memantine and Augmentin. Although the results were greater when Augmentin was given prior to scopolamine-induced AD, both Augmentin and memantine enhanced spatial memory in AD rats. In addition, PSEN2 and DDX5 were increased (but ITPKB was decreased) in the AD-treated group; however, the effects were more profound when combination therapy was used. Studies using flow cytometry found that pre-treatment with Augmentin decreased apoptosis in AD rats. This study showed that giving Augmentin to AD rats before memantine improved spatial memory, reduced neuronal cell death, upregulated protective genes, and suppressed genes involved in AD pathogenesis.

AD is a multifaceted disease that is influenced by environmental and genetic factors. These factors contribute to the onset or progression of AD, the most visible type of dementia seen in patients (Scheltens et al., 2021). AD currently affects approximately 15 million people worldwide, and this number is expected to rise to 80 million by 2040 (Javaid et al., 2021).

In this study, the group of rats given scopolamine showed marked impairment in memory. Scopolamine injection impaired spatial memory and caused neuronal cell death in AD rats (Kim et al., 2008; Tang, 2019). Scopolamine has been shown to activate serum inflammatory cytokines and inflammatory signaling pathways such as nuclear factor kappa B (NF-kB) (Iqbal et al., 2020). Increased acetylcholinesterase and decreased acetylcholine in the synaptic area, as well as oxidative stress and heightened cytotoxicity signals and levels of Aβ and free radicals, are all factors in the development of AD caused by scopolamine (Tang, 2019).

Treatment with memantine resulted in improved memory in AD rats. This suggests that this drug is a possible treatment for AD. Memantine inhibits glutamate production, which stops neuronal cell death and the development of AD. Cognitive impairment can be managed by memantine (Folch et al., 2018). The tolerance and safety parameters for memantine are well within acceptable ranges, making it a great treatment option. Memantine’s benefit lies in the fact that it only affects the channel when it is abnormally activated by an excess amount of glutamate in the synaptic cleft, as occurs in AD. Furthermore, preclinical data suggest that memantine may inhibit other receptors, such as nicotinic acetylcholine, serotonin, and sigma-1 (Lee et al., 2012; Allgaier and Allgaier, 2014). A study found that memantine improved cognitive function in rats in which ischemia had been induced (Lockrow et al., 2011). Another study found that memantine significantly reduced hippocampal nerve damage in rats with AD (Shafiei-Irannejad et al., 2021). It has also been demonstrated that treatment with a neuroprotective agent such as memantine can reduce the functional and morphological effects of ischemia on the brain (Tuo et al., 2022). Memantine was tested for its ability to mitigate the memory impairment caused by scopolamine in patients with amnesia, and it was shown to be effective (Dimitrova et al., 2021).

Augmentin (amoxicillin/clavulanic acid) is commonly used to treat infections of the urinary and respiratory tracts, ear, sinuses, and skin (Hakami et al., 2016). Previous research confirmed the role of Augmentin components (amoxicillin and clavulanic acid) in neurological disorders. In addition, recent research has demonstrated the potential of antibiotic-based therapy for treating and managing Parkinson’s disease and other neurodegenerative disorders (Yadav et al., 2021). In the case of amoxicillin, its administration has been reported to reduce epileptic seizures in patients with epilepsy and to reduce discrete body twitches (Raposo et al., 2016). The effects of clavulanic acid on Parkinson’s disease have shown that this medication shields hippocampal neurons from damage (Huh et al., 2010; Kost et al., 2012). Clavulanic acid’s effect on AD has also been investigated, and it was found to ameliorate behavioral abnormalities and protect against nerve cell death in AD patients (Huh et al., 2010). Although either amoxicillin alone or clavulanic acid alone has been investigated in a few studies, the role of Augmentin has not yet been determined. In this regard, our results for the first time revealed the protective effect of Augmentin in the pathogenesis of AD. This study showed that the effects of memantine alone were not as strong as when it was combined with Augmentin before scopolamine injection for AD induction. We believe that this effect might be due to a decrease in hippocampal cell apoptosis and a modulation of AD-associated gene expression.

PSEN2 is a critical component of *?*-secretase, which is responsible for the proteolytic cleavage of the amyloid precursor protein (APP) and the formation of Aβ peptides and has recently been linked to AD (Dong et al., 2022). Studies have found that AD-related PSEN2 dysregulation alters intracellular calcium signaling, resulting in the aggregation of Aβ to form brain plaques and cause neuronal cell death (Pizzo et al., 2020). DDX5 is significantly reduced in AD, and this inhibits ATP binding, cell proliferation and differentiation, and other activities and therefore may affect the underlying pathological aspects of AD (Li et al., 2021). ITPKB is upregulated in the cerebral cortex of patients with AD, increasing the development of amyloid plaques. Its upregulation has also increased the rate of neuronal cell death in AD patients, revealing that ITPKB could be a new target for AD treatment (Stygelbout et al., 2014). No studies demonstrated the relationship between the tested target genes and Augmentin and/or memantine. This study’s findings on the role of genes involved in AD pathogenesis were consistent with those of prior research. In this regard, the expression of the neuroprotective genes PSEN2 and DDX5 was enhanced, but the expression of ITPKB, a gene elevated in AD, tended to decline in our study when Augmentin was administered before memantine.

The activity of Augmentin in AD rats suggests that further extensive research on the utilization of ampicillin and clavulanic acid analogues in treating AD should be initiated. Virtual and high-throughput screening investigations of compound libraries, including pharmacophores derived from these parent compounds, may provide stronger results. Future perspectives are not limited to these areas of drug discovery; they should also be extended to the potential applications of Augmentin in clinical studies in AD patients. Augmentin might be a good candidate for improving memory function and decreasing neuronal cell apoptosis.

## 5 Conclusion

In this work, the effects of Augmentin on gene expression, cell apoptosis, and behavior were examined in scopolamine-induced AD rats. Memantine and Augmentin boosted spatial memory in rats. Although the effects of Augmentin on spatial memory in AD rats were greater when the drug was administered before the onset of the disease, both Augmentin and memantine improved memory in these animals. PSEN2 and DDX5 were upregulated, whereas ITPKB was downregulated, in the AD-treated group, with the effects being amplified by the use of the combination medication, indicating that the modulation of AD gene expression was possible with Augmentin treatment. Pre-treatment with Augmentin reduced apoptosis in AD rats, according to studies utilizing flow cytometry. Augmentin might be a valid addition to the AD treatment protocols.

## Data Availability

The original contributions presented in the study are included in the article/supplementary material, further inquiries can be directed to the corresponding author.

## Abbreviations

AD, Alzheimer’s disease; APP, the amyloid precursor protein; Aβ, amyloid-β; BDNF, Brain-derived neurotrophic factors; DDX5, DEAD-box helicase 5; GLT-1, glutamate transporter 1; ITPKB, inositol-trisphosphate 3-kinase B; MAPKs, mitogen-activated protein kinases; mTOR, the mammalian target of rapamycin; MMP, matrix metalloproteinases; NMDA, N-methyl-D-aspartate receptor; ROS, reactive oxygen species; NF-kB, nuclear factor kappa B; PCR, real-time quantitative reverse transcription PCR; PSEN2, presenilin-2; PI3K/PKB, phosphatidylinositol-3-kinase–protein kinase B; qRT-TREM2, triggering receptor expressed on myeloid cells 2.
